# A Retrospective Analysis of the Efficacy and Safety of Imatinib for Advanced Gastrointestinal Stromal Tumor in Elderly Patients

**DOI:** 10.1002/cam4.71338

**Published:** 2025-10-31

**Authors:** Takahito Awatsu, Hidekazu Hirano, Kengo Nagashima, Toshiharu Hirose, Natsuko Okita, Hirokazu Shoji, Atsuo Takashima, Akihito Nagahara, Ken Kato

**Affiliations:** ^1^ Department of Gastrointestinal Medical Oncology National Cancer Center Hospital Tokyo Japan; ^2^ Department of Gastroenterology Juntendo University, School of Medicine Tokyo Japan; ^3^ Biostatistics Unit, Clinical and Translational Research Center Keio University Hospital Tokyo Japan; ^4^ Department of Pathophysiological Research and Therapeutics for Gastrointestinal Disease Juntendo University, School of Medicine Tokyo Japan

**Keywords:** elderly, gastrointestinal stromal tumor, imatinib

## Abstract

**Background:**

Gastrointestinal stromal tumor (GIST) occurs predominantly in the elderly population. However, clinical data on the outcomes of treatment with imatinib in elderly patients are limited. The aim of this study was to determine the efficacy and safety of imatinib for advanced GIST in the elderly population compared with the non‐elderly population.

**Methods:**

We analyzed the clinical data of patients who received imatinib as first‐line treatment for advanced GIST at our hospital between January 2010 and July 2023. Progression‐free survival (PFS), overall survival (OS), and adverse events were compared between elderly patients (age ≥ 70 years, E group) and non‐elderly patients (age < 70 years, NE group).

**Result:**

Data of 91 patients (E group, *n* = 32; NE group, *n* = 59) with a median follow‐up of 39.8 months were analyzed. A significantly higher proportion of patients in the E group required a reduced starting dose of imatinib (34% vs. 2%, *p* < 0.001). Median PFS was 29.4 months in the E group and 60.0 months in the NE group (hazard ratio [HR] 2.04, *p* = 0.04, log‐rank test); median OS was 91.5 months in the E group and not reached in the NE group (HR 2.72, *p* = 0.03, log‐rank test). Multivariable analysis identified age as an independent prognostic factor for PFS (HR 2.26, *p* = 0.03) and a trend towards worse OS with increasing age (HR 2.93, *p* = 0.06). Grade 2 or higher non‐hematological adverse events were more prevalent in the E group (78% vs. 32%, *p* < 0.001).

**Conclusion:**

Imatinib is an effective treatment for advanced GIST in elderly patients. However, elderly patients were associated with poorer efficacy and a Higher incidence of toxicities due to imatinib compared with non‐elderly patients.

## 1 Introduction

Gastrointestinal stromal tumor (GIST) is a rare mesenchymal tumor that arises from the gastrointestinal tract and has an annual incidence rate of about 1–2 per 100,000 [1]. The treatment paradigm has progressed with the advent of imatinib and improved understanding of the molecular biological features of the disease. Imatinib is a tyrosine kinase inhibitor that competitively inhibits the ATP binding sites of receptor tyrosine kinases, such as KIT and platelet‐derived growth factor receptor A. Before the introduction of imatinib, cytotoxic agents were primarily used to treat advanced GIST but were associated with low response rates and a median overall survival (OS) of about 1 year [2]. Imatinib demonstrated significantly better efficacy for advanced GIST in pivotal clinical trials (B2222, S0033, EORTC 62005), with a median progression‐free survival (PFS) of about 20 months and a median OS of about 50 months [3, 4, 5]. Imatinib has been the standard first‐line treatment for advanced GIST for over 20 years.

GIST is more likely to occur in the elderly, and the median age at diagnosis is generally in the mid‐60s [1]. Elderly patients often have multiple comorbidities and diminished physical or cognitive function. Elderly patients with advanced GIST have been underrepresented in pivotal clinical trials with the median age of participants being approximately 60 years and only about 20% of patients being over 70 years old [3, 4, 5]. Several reports have provided insight into clinical practice for elderly patients with GIST. Surgery and adjuvant imatinib are less likely to be offered for localized GIST in elderly patients [6]. Furthermore, imatinib tends to be administered at a lower dosage in elderly patients than in their younger counterparts [7, 8]. Whether there is a difference in real‐world clinical outcomes of treatment of advanced GIST with imatinib between elderly and non‐elderly patients remains controversial [6, 7, 8, 9].

Given the lack of sufficient data on elderly patients with advanced GIST treated by imatinib, further exploration is warranted to better understand treatment outcomes in this age group in clinical practice. The aim of this study was to determine the efficacy and safety of imatinib for advanced GIST in elderly patients compared with nonelderly patients.

## 2 Methods

### 2.1 Patient Population

We analyzed clinical data of patients with advanced GIST treated with imatinib as first‐line treatment at our hospital between January 2010 and July 2023. The selection criteria were as follows: age 20 years or older; Eastern Cooperative Oncology Group performance status of 0–2; initially unresectable or recurrent disease; and histologically confirmed GIST. Patients who had recurrence during adjuvant imatinib treatment or within 6 months of completing adjuvant imatinib treatment were excluded. The study was approved by the Institutional Review Board of the National Cancer Center Hospital (approval number: 2017‐229). Informed consent was obtained from all patients via the opt‐out route.

### 2.2 Assessment

The data collected included clinicopathological characteristics, details of imatinib administration, and treatment outcomes. Contrast‐enhanced or plain computed tomography scans were generally performed every 3–4 months. PFS was calculated as the interval from the date of initiation of imatinib to disease progression or death or censored at the latest follow‐up for surviving patients without disease progression or cytoreductive surgery. OS was calculated as the interval between the date of initiation of imatinib and death or censored at the latest follow‐up for surviving patients. Tumor responses were assessed according to Response Evaluation Criteria in Solid Tumors (RECIST) version 1.1. Adverse events (AEs) were extracted and collected from medical records evaluated by each physician based on the Common Terminology Criteria for Adverse Events (CTCAE) version 5.0. The cut‐off date for clinical outcomes was August 31, 2023.

### 2.3 Treatment

Imatinib was administered once daily at a dose of 400 mg or 300 mg or lower. Treatment was continued until disease progression, unacceptable toxicity, or death. Generally, dose interruption was implemented at the physician's discretion when grade ≥ 3 hematologic AEs or grade ≥ 2 non‐hematologic AEs were observed. Dose reduction was performed when grade 4 or prolonged grade 3 hematologic AEs, or grade ≥ 3 or prolonged grade 2 non‐hematologic AEs, were observed, according to the physician's judgment.

### 2.4 Statistical Analysis

The patients were divided into an elderly group (started on imatinib for advanced GIST at ≥ 70 years of age, E group) and a non‐elderly group (started on imatinib for advanced GIST at < 70 years of age, NE group). Quantitative data are expressed as the median with the 95% confidence interval (CI) or range. The Mann–Whitney *U* test was used for between‐group comparisons of continuous variables and the chi‐squared test or Fisher's exact test, as appropriate, for categorical variables. PFS and OS were analyzed by the Kaplan–Meier method, with the log‐rank test used for between‐group comparisons. Hazard ratios (HRs) were calculated by using a Cox proportional hazards model. The effects of independent variables on OS and PFS were assessed through univariable and multivariable analyses using Cox proportional hazards models. Age, sex, and Eastern Cooperative Oncology Group performance status as well as primary site, tumor diameter, disease status, and *KIT* mutation status were selected as covariates for univariable analysis based on previous reports [3, 4, 5, 10, 11]. Covariates with a *p*‐value of < 0.10 in univariable analysis were used in multivariable analyses. Univariable and multivariable analyses were performed for patients with no missing data for *KIT* mutation status. All tests were two‐sided, with *p*‐values of < 0.05 considered statistically significant. Multiplicity was not adjusted for because of the exploratory nature of the study. All statistical analyses were conducted using R version 4.3.2 and EZR software for Windows version 1.55 [12].

## 3 Results

Data of 91 patients (E group, *n* = 32; NE group, *n* = 59) were analyzed. The patients' clinicopathological characteristics are shown in Table 1. No significant differences were found between the E group and the NE group except for the proportions with a performance status of 0 (19% vs. 63%, *p* < 0.001) and a reduced starting dose of imatinib (34% vs. 2%, *p* < 0.001). *KIT* exon 11 mutations were identified in 50% of patients in the E group and 61% in the NE group. Information on *KIT* mutation status was missing in 6 patients (20%) in the E group and 10 (17%) in the NE group. All patients had at least 1 measurable lesion. The median follow‐up duration was 39.8 months (95% CI 28.2–53.1).

**TABLE 1:** Baseline characteristics of patients who received imatinib as first‐line treatment.

	All patients (*n* = 91)	Elderly (*n* = 32)	Non‐elderly (*n* = 59)	*p*
Male sex	49 (54)	16 (50)	33 (56)	0.66
Median age, years (range)	64 (29–90)	75.5 (70–90)	54 (29–69)	
Median BMI, kg/m^2^ (range)	21.6 (15.6–30.7)	21.9 (15.6–30.7)	21.4 (16.9–29.4)	0.6
Performance status, *n* (%)				< 0.001
0	43 (47)	6 (19)	37 (63)	
1	42 (46)	22 (69)	20 (34)	
2	6 (7)	4 (13)	2 (3)	
Disease status, *n* (%)				0.83
Initially unresectable	41 (45)	15 (47)	26 (44)	
Recurrent	50 (55)	17 (53)	33 (56)	
Initial imatinib dose, *n* (%)				< 0.001
400 mg/day	79 (87)	21 (66)	58 (98)	
≤ 300 mg/day	12 (13)	11 (34)	1 (2)	
*KIT* mutation, *n* (%)				0.21
Exon 11	51 (56)	16 (50)	36 (61)	
Exon 9	14 (15)	4 (13)	10 (17)	
Others^a^	9 (10)	6 (20)	3 (5)	
Missing	16 (18)	6 (20)	10 (17)	
Primary site, *n* (%)				0.69
Stomach	32 (35)	13 (41)	19 (32)	
Small bowel	45 (49)	14 (44)	31 (53)	
Others^b^	14 (15)	5 (16)	9 (15)	
Metastasis site, *n* (%)				
Liver	48 (53)	16 (50)	33 (56)	0.48
Peritoneum	60 (66)	23 (72)	37 (63)	0.23
Maximum tumor diameter, *n* (%)				0.19
< 8 cm	40 (44)	11 (34)	29 (49)	
≥ 8 cm	51 (56)	21 (66)	30 (51)	

Abbreviations: BMI, body mass index; *PDGFRA*, platelet‐derived growth factor receptor A.

^a^ *PDGFRA* exon 18 mutation (*n* = 1), wild‐type (*n* = 8).

^b^ Colon and rectum (*n* = 7), esophagus (*n* = 2), peritoneum (*n* = 5).

### 3.1 Efficacy

**FIGURE 1:**
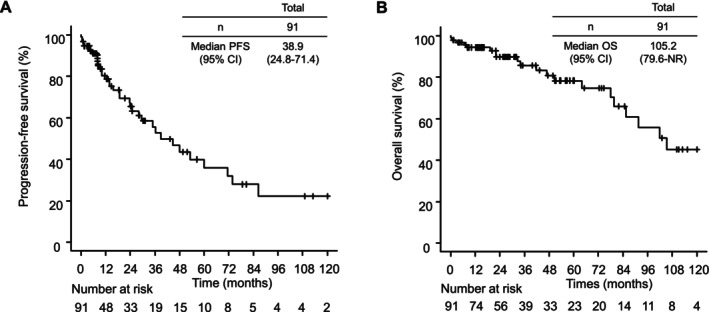
Kaplan–Meier survival curves for the overall population. (A) Progression‐free survival. (B) Overall survival.

In the overall study population, median PFS was 38.9 months (95% CI 24.8–71.4) (Figure 1A) and median OS was 105.2 months (95% CI 79.6–not reached) (Figure 1B).

**FIGURE 2:**
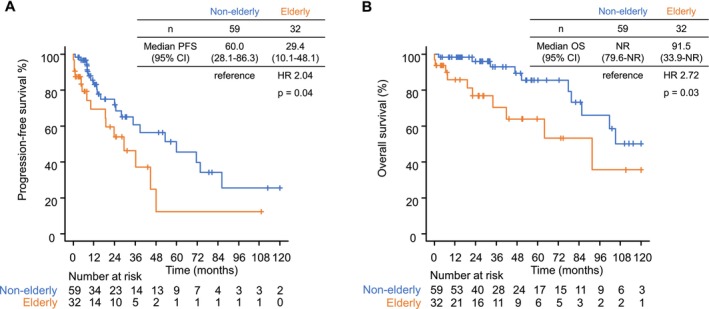
Kaplan–Meier survival curves according to age group. (A) Progression‐free survival. (B) Overall survival.

Median PFS was 29.4 months (95% CI 10.1–48.1) in the E group and 60.0 months (95% CI 28.1–86.3) in the NE group (HR 2.04, 95% CI 1.04–4.02, *p* = 0.04) (Figure 2A). The respective 1‐year, 3‐year, and 5‐year PFS rates were 69.3%, 46.3%, and 12.3% in the E group and 83.2%, 60.6%, and 51.2% in the NE group. The results of univariable and multivariable analyses for PFS are shown in Table 2. Multivariable analysis revealed age to be an independent prognostic factor for PFS (*p* = 0.007) and that PFS was worse when the primary site was outside the stomach (*p* = 0.03) and when GIST was initially unresectable (*p* < 0.001).

**TABLE 2:** Results of univariable and multivariable analyses for progression‐free survival.

	Univariable analysis		Multivariable analysis	
	HR (95% CI)	*p*	HR (95% CI)	*p*
Sex (female vs. male [ref])	1.47 (0.74–2.94)	0.27		
Age (elderly vs. non‐elderly [ref])	2.26 (1.08–4.71)	0.03	3.17 (1.38–7.32)	0.007
Performance status (1–2 vs. 0 [ref])	3.28 (1.51–7.11)	0.003	2.07 (0.82–5.21)	0.12
Disease status (initially unresectable vs. recurrent [ref])	8.43 (2.96–24.03)	< 0.001	13.72 (3.66–51.37)	< 0.001
*KIT* mutation (other than exon 11 vs. exon 11 [ref])	4.03 (2.01–8.08)	< 0.001	2.12 (0.93–4.85)	0.07
Primary site (other than stomach vs. stomach [ref])	2.74 (1.17–6.41)	0.02	3.05 (1.14–8.20)	0.03
Maximum tumor diameter (≥ 8 cm vs. < 8 cm [ref])	4.55 (2.01–10.30)	< 0.001	1.63 (0.65–4.08)	0.29
Initial dose reduction (present vs. absent [ref])	1.79 (0.67–4.73)	0.24		

*Note:* Univariable and multivariable analyses were performed for 75 cases with available KIT mutation status.

Abbreviations: CI, confidence interval; HR, hazard ratio; Ref, reference.

Median OS was 91.5 months (95% CI 33.9–not reached) in the E group and not reached (95% CI 79.6–not reached) in the NE group (HR 2.72, 95% CI 1.13–6.59, *p* = 0.03) (Figure 2B). The respective 1‐year, 3‐year, and 5‐year OS rates were 85.8%, 70.3%, and 63.9% in the E group and 98.3%, 92.9%, and 85.4% in the NE group. The results of the univariable and multivariable analyses for OS are shown in Table 3. Older age was a significant independent poor prognostic factor for OS in univariable analysis (*p* = 0.03) and tended to be associated with a poor prognosis in multivariable analysis (*p* = 0.06).

**TABLE 3:** Results of univariable and multivariable analyses for overall survival.

	Univariable analysis		Multivariable analysis	
	HR (95% CI)	*p*	HR (95% CI)	*p*
Sex (female vs. male [ref])	0.61 (0.23–1.67)	0.34		
Age (elderly vs. non‐elderly [ref])	2.98 (1.14–7.81)	0.03	2.93 (0.95–9.01)	0.06
Performance status (1–2 vs. 0 [ref])	2.82 (1.08–7.36)	0.03	1.79 (0.52–6.14)	0.36
Disease status (initially unresectable vs. recurrent [ref])	2.67 (0.86–8.30)	0.09	2.89 (0.74–11.23)	0.13
*KIT* mutation (other than exon 11 vs. exon 11 [ref])	3.51 (1.33–9.25)	0.01	2.94 (0.92–9.37)	0.07
Primary site (other than stomach vs. stomach [ref])	2.64 (0.85–8.22)	0.09	2.85 (0.79–10.27)	0.11
Maximum tumor diameter (≥ 8 cm vs. < 8 cm [ref])	2.92 (1.05–8.13)	0.04	1.68 (0.50–5.70)	0.4
Initial dose reduction (present vs. absent [ref])	2.54 (0.53–12.09)	0.24		

*Note:* Univariable and multivariable analyses were performed for 75 cases with available KIT mutation status.

Abbreviations: CI, confidence interval; HR, hazard ratio; Ref, reference.

The objective response rate was 60% in the overall population, with no significant differences between the E group and the NE group (53% vs. 66%, *p* = 0.26). The disease control rate was significantly higher in the NE group (91% vs. 100%, *p* = 0.04) (Table 4).

**TABLE 4:** Best overall response.

	Total	Elderly	Non‐elderly	*p*
	(*n* = 91)	(*n* = 32)	(*n* = 59)	
	*n* (%)	*n* (%)	*n* (%)	
Complete response (CR)	4 (4)	2 (6)	2 (3)	
Partial response (PR)	52 (57)	15 (47)	37 (63)	
Stable disease (SD)	33 (36)	12 (38)	20 (34)	
Progressive disease (PD)	3 (3)	3 (9)	0 (0)	
Objective response rate (CR + PR)	55 (60)	17 (53)	39 (66)	0.26
Disease control rate (CR + PR + SD)	88 (97)	29 (91)	59 (100)	0.04

### 3.2 Safety and Toxicity

The frequencies of AEs during treatment with imatinib are presented in Table 5. There was no significant difference in any grade 3 or higher hematological AEs between the E group and the NE group (28% vs. 27%, *p* = 1). Any grade 3 or higher non‐hematological AEs were more common in the E group than in the NE group, but not significantly (34% vs. 17%, *p* = 0.11). Grade 2 or higher non‐hematological AEs were significantly more frequent in the E group than in the NE group (78% vs. 32%, *p* < 0.001). In contrast, any grade 2 or higher hematological AEs were significantly more common in the NE group (41% vs. 66%, *p* = 0.03). Among the grade 2 or higher non‐hematological AEs, creatinine increased (22% vs. 5%, *p* = 0.03), fluid retention (41% vs. 15%, *p* = 0.01), and hypertension (9% vs. 0%, *p* = 0.04) were significantly more common in the E group than in the NE group. The proportion of patients in whom the initial dose of imatinib was reduced from 400 mg/day was significantly higher in the E group (76% vs. 45%, *p* = 0.03). The median time to dose reduction from starting dose was 69 days (range: 11–4285). The proportion of dose reduction in the population who started at a reduced dose (≤ 300 mg/day) was higher in the E group (63% vs. 0%). Among elderly patients, there were no significant differences in PFS and OS between those with initial dose reduction and those without initial dose reduction (Figure S1A; Figure 1B). Among patients having dose reduction after treatment initiation and PFS events, the post‐reduction duration was significantly longer than the pre‐reduction duration (Figure S2). Three patients (9%) in the E group and 2 (3%) in the NE group discontinued imatinib because of AEs; the between‐group difference was not significant (*p* = 0.34). In addition, the characteristics of the elderly group by initial dose of imatinib are presented in Table S1. Among elderly patients, patients who started with reduced initial doses had a significantly higher age (*p* < 0.001) and larger maximum tumor diameter (*p* < 0.001) than those who started with standard doses.

**TABLE 5:** Adverse events during treatment with imatinib.

	Any grade, *n* (%)		*p*	Grade ≥ 2 (%)		*p*	Grade ≥ 3, *n* (%)		*p*
	Elderly	Non‐elderly		Elderly	Non‐elderly		Elderly	Non‐elderly	
	(*n* = 32)	(*n* = 59)		(*n* = 32)	(*n* = 59)		(*n* = 32)	(*n* = 59)	
Any hematological AE	24 (75)	54 (92)	0.06	13 (41)	39 (66)	0.03	9 (28)	16 (27)	1
Anemia	23 (72)	47 (80)	0.56	12 (38)	29 (49)	0.4	6 (19)	8 (14)	0.73
Neutropenia	7 (22)	34 (58)	0.002	6 (19)	24 (41)	0.06	5 (16)	9 (15)	1
Thrombocytopenia	12 (38)	21 (36)	1	3 (9)	4 (7)	0.69	3 (9)	2 (3)	0.34
Any non‐hematological AE	32 (100)	58 (98)	1	25 (78)	19 (32)	< 0.001	11 (34)	10 (17)	0.11
AST elevation	18 (56)	46 (78)	0.05	4 (13)	10 (17)	0.76	2 (6)	5 (8)	1
ALT elevation	17 (53)	34 (58)	0.85	4 (13)	9 (15)	1	2 (6)	4 (7)	1
Creatinine increased	22 (69)	32 (54)	0.26	7 (22)	3 (5)	0.03	1 (3)	1 (2)	1
Nausea	9 (25)	12 (20)	0.56	3 (9)	1 (2)	0.12	1 (3)	0 (0)	0.35
Anorexia	12 (34)	16 (27)	0.43	5 (16)	3 (5)	0.12	0 (0)	1 (2)	1
Diarrhea	11 (34)	22 (37)	0.96	1 (3)	7 (12)	0.25	0 (0)	0 (0)	NA
Fatigue	11 (34)	15 (25)	0.51	2 (6)	1 (2)	0.28	0 (0)	0 (0)	NA
Fluid retention	25 (78)	36 (61)	0.15	13 (41)	9 (15)	0.01	5 (16)	5 (8)	0.31
Skin toxicity	13 (41)	12 (20)	0.07	4 (13)	2 (3)	0.18	0 (0)	1 (2)	1
Hypertension	3 (9)	0 (0)	0.04	3 (9)	0 (0)	0.04	0 (0)	0 (0)	NA
Myalgia	5 (16)	14 (24)	0.43	2 (6)	1 (2)	0.28	0 (0)	0 (0)	NA
Perforation	1 (3)	1 (2)	1	1 (3)	1 (2)	1	1 (3)	1 (2)	1
Pneumonitis	1 (3)	0 (0)	0.35	1 (3)	0 (0)	0.35	1 (3)	0 (0)	0.35

Abbreviations: AE, adverse event; ALT, alanine transaminase; AST, aspartate transaminase; NA, not available.

### 3.3 Cytoreductive Surgery Following First‐Line Imatinib Treatment

Cytoreductive surgery was performed following imatinib in 4 patients (13%) in the E group and 16 patients (27%) in the NE group (*p* = 0.12). R0 resection was achieved in all patients who underwent cytoreductive surgery with the exception of 1 case of R2 resection in the E group. One patient (25%) in the E group and 4 (25%) in the NE group had tumor progression after cytoreductive surgery (*p* = 1).

### 3.4 Post‐Progression Treatment

Fifteen patients in the E group and 26 in the NE group had disease progression after imatinib treatment with or without cytoreductive surgery. The proportion of patients who received post‐progression treatment was significantly lower in the E group than in the NE group (40% vs. 88%, *p* = 0.003). The reasons for not receiving post‐progression treatment were deterioration in general condition (6 patients in the E group) and patient refusal (3 patients in the E group and 3 patients in the NE group). Among patients receiving post‐progression treatment, 6 patients (100%) in the E group and 19 (83%) in the NE group were subsequently treated with sunitinib and the remaining 4 patients in the NE group received investigational agents in clinical trials. The median PFS of sunitinib as second‐line treatment was 25.5 months (95% CI 0.6–NR) in the E group and 8.0 months (95% CI 4.3–17.3) in the NE group (HR 0.39, 95% CI 0.10–1.48, *p* = 0.17) (Figure S3A). The median OS of sunitinib as second‐line treatment was 34.3 months (95% CI 5.7–NR) in the E group and 41.3 months (95% CI 24.3–NR) in the NE group (HR 1.43, 95% CI 0.41–5.02, *p* = 0.57) (Figure S3B).

## 4 Discussion

This investigation had several important findings. First, outcomes were worse in elderly patients who received imatinib for advanced GIST than in their non‐elderly counterparts. Second, the proportion of patients in whom the starting dose of imatinib was reduced and the incidence of grade 2 or higher non‐hematologic AEs were significantly higher in the elderly population than in the non‐elderly population. These findings suggest that, in clinical practice, elderly patients with advanced GIST are disadvantaged in terms of treatment with imatinib. This is the first study to provide an in‐depth assessment of treatment outcomes, including safety considerations, and details of subsequent treatment for elderly patients with advanced GIST who are treated with imatinib in Japan.

The results of our study indicate that the survival benefit conferred by imatinib for advanced GIST is smaller in elderly patients than in non‐elderly patients. Previous studies of the prognostic impact of age in patients with advanced GIST have yielded conflicting results [6, 7, 9, 13]. In studies focusing on elderly GIST patients, including our own, differences exist in the definition of elderly patients, geographical variability, and the study period. Given these factors, differences in general condition, the impact of comorbidities, pharmacokinetics, imatinib administration strategies (e.g., initial dose reduction) may have collectively influenced treatment outcomes in elderly GIST patients. A strength of our study is that it focuses on patients who have received treatment in recent years and investigates the associations between age groups and OS using multivariable analyses, revealing a trend of poorer OS in elderly patients (HR 2.93, 95% CI 0.95–9.01; *p* = 0.06). This trend may be explained by inferior PFS and a lower frequency of post‐progression treatment. Previous Rutkowski's study showed no obvious difference in terms of PFS in elderly patients compared with non‐elderly patients with the same definition of elderly patients (≥ 70 years) [7]. On the other hand, our study showed a trend towards inferior progression‐free survival in elderly patients. In addition, compared to our study, Rutkowski's study reported a lower dose reduction rate of imatinib (16.9%) and a lower incidence of Grade 3/4 adverse events (16.5%). Patients in Rutkowski's study experienced fewer toxicities, which may have allowed them to receive more intensive imatinib treatment, potentially leading to better progression‐free survival. This discrepancy between study results might be attributed to ethnic differences. Asian patients are more likely to experience imatinib‐related adverse events [14]. The metabolic enzymes involved in imatinib metabolism exhibit ethnic differences (e.g., ABCG2 421C>A allele is more prevalent in the Asian population), and the blood concentration of imatinib tends to be higher in Asians than in Caucasians. Since adverse events related to imatinib are more frequent in Asians, treatment intensity may be reduced, particularly in elderly patients, potentially leading to decreased antitumor efficacy.

OS seems to be superior in our study population than in the previous pivotal trials, which is in line with recent reports [3, 4, 5, 10, 15]. This finding could be attributed to the upfront use of imatinib and availability of additional drug therapies after imatinib [16, 17, 18, 19, 20]. Furthermore, several retrospective and prospective studies have indicated that cytoreductive surgery may confer a survival benefit in patients with advanced GIST who respond to imatinib [15, 21, 22, 23]. In our study, cytoreductive surgery was more likely to be performed in non‐elderly patients than in elderly patients (27% vs. 13%). Although the between‐group difference in the proportion of patients who underwent cytoreductive surgery was slight, it may have contributed to the disparity in survival rates between the two groups.

In our study, we focused not only on AEs of any grade or those of grade 3 or higher but also on AEs of grade 2 or higher. According to CTCAE definitions, grade 2 or higher AEs are considered to cause limitations in activities of daily living and to require therapeutic intervention. In our study, non‐hematological AEs of grade 2 or higher (e.g., creatinine increased, fluid retention, and hypertension) were more common in elderly patients. Previous reports on the safety of imatinib in patients with advanced GIST indicate a trend towards a higher incidence of grade 3 or higher AEs among the elderly population [6, 7]. Our study shows a similar trend, particularly for non‐hematological AEs. Other studies indicate that the starting doses of imatinib are lower in elderly patients than in non‐elderly patients in Asian populations but not in the European population [8]. We observed a similar trend of elderly patients being started on imatinib at a dose that was lower than that in younger patients. In our institution, the proportion of patients with initial dose reduction among elderly patients with advanced GIST was higher in more elderly patients and those with a higher tumor burden (Table S1), supporting the fact that dose adjustment was performed in consideration of the patients' condition. Furthermore, to evaluate the impact of initial dose reduction on survival outcomes in elderly patients, we conducted a survival analysis for each initial dose (Figure S1). As a result, there was no difference in either PFS or OS (PFS: HR 1.42, *p* = 0.54; OS: HR 1.94, *p* = 0.37), and the results for OS were consistent with previous reports [13]. Notably, more than half of our elderly patients who started imatinib at a dose < 300 mg/day required further dose reduction, primarily because of AEs. Previous studies have noted a similar trend of dose reduction during the administration of imatinib owing to AEs in elderly patients [7, 24]. Farag et al. reported that AEs resulted in discontinuation of imatinib in more than half of their elderly population treated with imatinib at a mean dose of 400 mg/day [6]. Although the diseases differed, the result of a retrospective study on dose adjustment and clinical outcomes of imatinib in elderly patients with chronic myeloid leukemia (CML) supported the findings of this study [25]. The low imatinib discontinuation rate in this study may be due to the careful dose adjustment at initiation and during treatment, which is particularly important in elderly patients and has a positive effect on survival outcomes.

In this study, elderly patients were less likely to receive post‐progression treatment after imatinib failure than non‐elderly patients, mainly because of deterioration in their general condition. The current standard second‐line treatment for advanced GIST is sunitinib [26]. Sunitinib tends to have a high toxicity profile, with a reported incidence of grade 3–4 AEs of 56% in the real‐world setting [27]. It has also been reported that advanced age is a risk factor for the development of non‐hematological grade 3 or higher AEs, dose reduction, dose interruption, and permanent discontinuation in patients receiving sunitinib [23, 27, 28, 29, 30]. The findings of our study may reflect the tendency for sunitinib to be introduced cautiously in elderly patients after failure of imatinib, considering its high toxicity profile in the real‐world setting. Although the sample size was small, the results suggested that elderly patients who were able to receive sunitinib in clinical practice may have comparable PFS as compared with non‐elderly patients. Given the small number of elderly patients receiving sunitinib, the development of second‐line drugs that are better tolerated and improve outcomes is needed in this population whose general condition is deteriorating.

Although elderly patients tend to have poorer physiological function, chronological age alone should not be the primary factor determining treatment, given that physiological function varies among individuals [31]. It is important to formulate a treatment plan while considering aging‐related problems in individual patients. The American Society of Clinical Oncology recommends geriatric assessment (GA) for elderly patients who are undergoing chemotherapy to identify vulnerabilities that are typically overlooked in standard oncology evaluations [32]. It has been reported that GA improves satisfaction with treatment among patients and caregivers and that it is associated with fewer severe AEs resulting from cancer treatment [33, 34]. Nevertheless, the clinical value of GA in elderly patients with advanced GIST has not been fully established and requires additional investigation. Pharmacokinetics are influenced by both endogenous and exogenous factors, including age, sex, genetic polymorphisms, performance status, comorbidities, hepatic and renal function, and concomitant medications, leading to interindividual variability. The standard dose of imatinib is 400 mg/day irrespective of clinical parameters, such as body weight and body surface area. However, given that AEs are more common in elderly patients than in non‐elderly patients, it is important to tailor the dose of imatinib to the individual elderly patient. Moreover, there is concern that unnecessary dose reductions could diminish the effectiveness of imatinib in elderly patients who are able to tolerate standard doses. Determining the dosage of imatinib based on therapeutic drug monitoring (TDM) may be useful for optimization of the balance between treatment response and AEs, as suggested in previous studies [35, 36, 37, 38, 39, 40]. Development of personalized management is crucial for elderly patients, incorporating careful determination of the initial dose, comprehensive assessment of comorbidities and performance status, early management of AEs with dose adjustments as needed, support for treatment continuation, and utilization of CGA and TDM. In CML, intermittent imatinib administration has been suggested as a viable strategy for elderly patients who have achieved sustained disease control, offering the potential to maintain efficacy while reducing AEs. However, further investigation is warranted to determine whether a similar approach could be effective in the management of GIST [41].

This study has several limitations. First, it had a retrospective single‐center design and a limited number of Japanese patients, which may affect the generalizability of its findings. Second, some clinical data were missing, including for *KIT* mutation status. Third, the clinicopathological characteristics at baseline were not balanced between the two groups. Although multivariable analysis was performed to mitigate these imbalances, confounding factors may still exist. Fourth, information on AEs was collected from the medical records in retrospective studies, which may result in underreporting or omission of details and causal relationships, particularly for lower‐grade AEs. Fifth, due to the retrospective nature of the study, there is a lack of detailed information on supportive care for AEs caused by imatinib. The findings of this study should be further investigated through large‐scale, multicenter international studies to address these limitations. In addition, it is desirable to design prospective observational studies based on the information obtained from these studies, and to realize personalized treatment for elderly GIST patients.

## 5 Conclusion

Imatinib serves as an effective treatment for advanced GIST in elderly patients, yet it demonstrates poorer efficacy and a higher rate of toxicities in elderly patients compared with nonelderly patients.

## Author Contributions

**Takahito Awatsu:** data curation (lead), formal analysis (lead), investigation (lead), methodology (lead), resources (lead), supervision (equal), validation (lead), visualization (lead), writing – original draft (lead), writing – review and editing (equal). **Hidekazu Hirano:** conceptualization (lead), data curation (supporting), formal analysis (supporting), investigation (supporting), methodology (supporting), resources (supporting), supervision (lead), validation (supporting), visualization (supporting), writing – original draft (supporting), writing – review and editing (lead). **Toshiharu Hirose:** validation (supporting), writing – review and editing (supporting). **Natsuko Okita:** validation (supporting), writing – review and editing (supporting). **Hirokazu Shoji:** validation (supporting), writing – review and editing (supporting). **Atsuo Takashima:** validation (supporting), writing – review and editing (supporting). **Akihito Nagahara:** validation (supporting), writing – review and editing (supporting). **Ken Kato:** project administration (lead), validation (supporting), writing – original draft (supporting), writing – review and editing (supporting). **Kengo Nagashima:** methodology (equal).

## Ethics Statement

This study was approved by the Institutional Review Board of the National Cancer Center Hospital, Japan (approval number: 2017–229). This study was conducted in accordance with the ethical principles outlined in the Declaration of Helsinki.

## Consent

Patient consent was obtained through an opt‐out method.

## Conflicts of Interest

Author H.H. has received research grants and speaker honoraria from Novartis, Ono Pharmaceutical, Taiho Pharmaceutical, Bristol‐Myers Squibb, PPD, Daiichi‐Sankyo, Nippon Boehringer Ingelheim, ALX Oncology, BeiGene, and Amgen. Author T.H. has received research grants and speaker honoraria from Ono Pharmaceutical, Taiho Pharmaceutical, Bristol‐Myers Squibb, and Lilly. Author A.T. received research grants and speaker honoraria from Ono Pharmaceutical, Taiho Pharmaceutical, Bristol‐Myers Squibb, Lilly, Takeda, Chugai Pharma, Merck Serono, Merck Sharp & Dohme, Amgen, AstraZeneca, and Eisai. Author K.K. has received research grants and speaker honoraria from Merck Sharp & Dohme, Ono Pharmaceutical, Bristol Myers Squibb, BeiGene, Shionogi, Merck Biopharma, Oncolys BioPharma, Daiichi Sankyo, Novartis, Taiho Pharmaceutical, Janssen, AstraZeneca, and Chugai. The other authors declare no conflicts of interest.

## Supporting information


**Table S1.** Characteristics of elderly patients by initial dose of imatinib.

**Figure S1.** Kaplan–Meier survival curves for initial dose of imatinib in elderly population. (A) Progression‐free survival. (B) Overall survival.

**Figure S2.** Comparison of the treatment duration before and after imatinib dose reduction.

**Figure S3.** Kaplan–Meier survival curves of sunitinib as second‐line treatment according to age group. (A) Progression‐free survival. (B) Overall survival.

## Data Availability

All data generated or analyzed during this study is included in this published article.
