# Supplementary material for: A Retrospective Analysis of the Efficacy and Safety of Imatinib for Advanced Gastrointestinal Stromal Tumor in Elderly Patients
Source: Cancer Med. 2025 Oct 31;14(21):e71338. doi: 10.1002/cam4.71338 (PMC12576807; doi:10.1002/cam4.71338)
Supplement: Supplementary file 1 — Table S1. Characteristics of elderly patients by initial dose of imatinib. [file CAM4-14-e71338-s004.docx]

**Supplementary Materials**

**Table**

Supplementary Table 1. Characteristics of elderly patients by initial dose of imatinib

|  | Elderly patients (n=32) | Initial dose of 400 mg/day (n=21) | Initial dose of ≤ 300 mg/day  (n=11) | *p*-Value |
| --- | --- | --- | --- | --- |
| Sex (male), n (%) | 16 (50) | 11 (52) | 5 (45) | 1 |
| Median age, years (range) | 75.5 (70-90) | 73 (70-88) | 83 (72-90) | < 0.001 |
| Median BMI (kg/m^2^) (range) | 21.9 (15.6-30.7) | 22.1 (18.6-30.7) | 21.4 (16.9-29.4) | 0.1 |
| Performance Status, n (%) |  |  |  | 0.20 |
| 0 | 6 (19) | 5 (24) | 1 (9) |  |
| 1 | 22 (81) | 15 (71) | 7 (64) |  |
| 2 | 4 (13) | 1 (5) | 3 (27) |  |
| Disease status, n (%) |  |  |  | 0.71 |
| Initially unresectable | 15 (47) | 9 (43) | 6 (54) |  |
| Recurrent | 17 (53) | 12 (57) | 5 (46) |  |
| *KIT* mutation*,* n (%) |  |  |  | 0.31 |
| Exon 11 | 16 (50) | 9 (43) | 7 (64) |  |
| Exon 9 | 4 (13) | 4 (19) | 0 (0) |  |
| Others^†^ | 6 (20) | 5 (24) | 1 (9) |  |
| Missing | 6 (20) | 3 (14) | 3 (27) |  |
| Primary site, n (%) |  |  |  | 0.68 |
| Stomach | 13 (35) | 6 (29) | 7 (64) |  |
| Small bowel | 14 (49) | 11 (52) | 3 (27) |  |
| Others^‡^ | 5 (15) | 4 (19) | 1 (9) |  |
| Metastasis site, n (%) |  |  |  |  |
| Liver | 16 (50) | 11 (52) | 5 (46) | 0.48 |
| Peritoneum | 23 (72) | 16 (76) | 7 (64) | 0.09 |
| Maximum tumor diameter, n (%) |  |  |  | < 0.001 |
| < 8cm | 11 (34) | 9 (43) | 2 (18) |  |
| ≥ 8cm | 21 (66) | 12 (57) | 9 (82) |  |

^†^*PDGFRA* exon 18 mutation (n = 1), wild-type (n = 5). ^‡^Colon and rectum (n = 3), esophagus (n = 1), peritoneum (n = 1). Abbreviations: BMI, body mass index; *PDGFRA*, platelet-derived growth factor receptor A

**Figure legends**

- ***Supplementary Figure 1***. Kaplan–Meier survival curves for initial dose of imatinib in elderly population
- A: Progression-free survival
- B: Overall survival
- ***Supplementary Figure 2.*** Comparison of the treatment duration before and after imatinib dose reduction
- ***Supplementary Figure 3.*** Kaplan–Meier survival curves of sunitinib as second-line treatment according to age group
- A: Progression-free survival
- B: Overall survival
