# Supplementary material for: A Retrospective Analysis of the Efficacy and Safety of Imatinib for Advanced Gastrointestinal Stromal Tumor in Elderly Patients
Source: Cancer Med. 2025 Oct 31;14(21):e71338. doi: 10.1002/cam4.71338 (PMC12576807; doi:10.1002/cam4.71338)

Supplementary Figure 1. Kaplan–Meier survival curves for initial dose of imatinib in elderly population

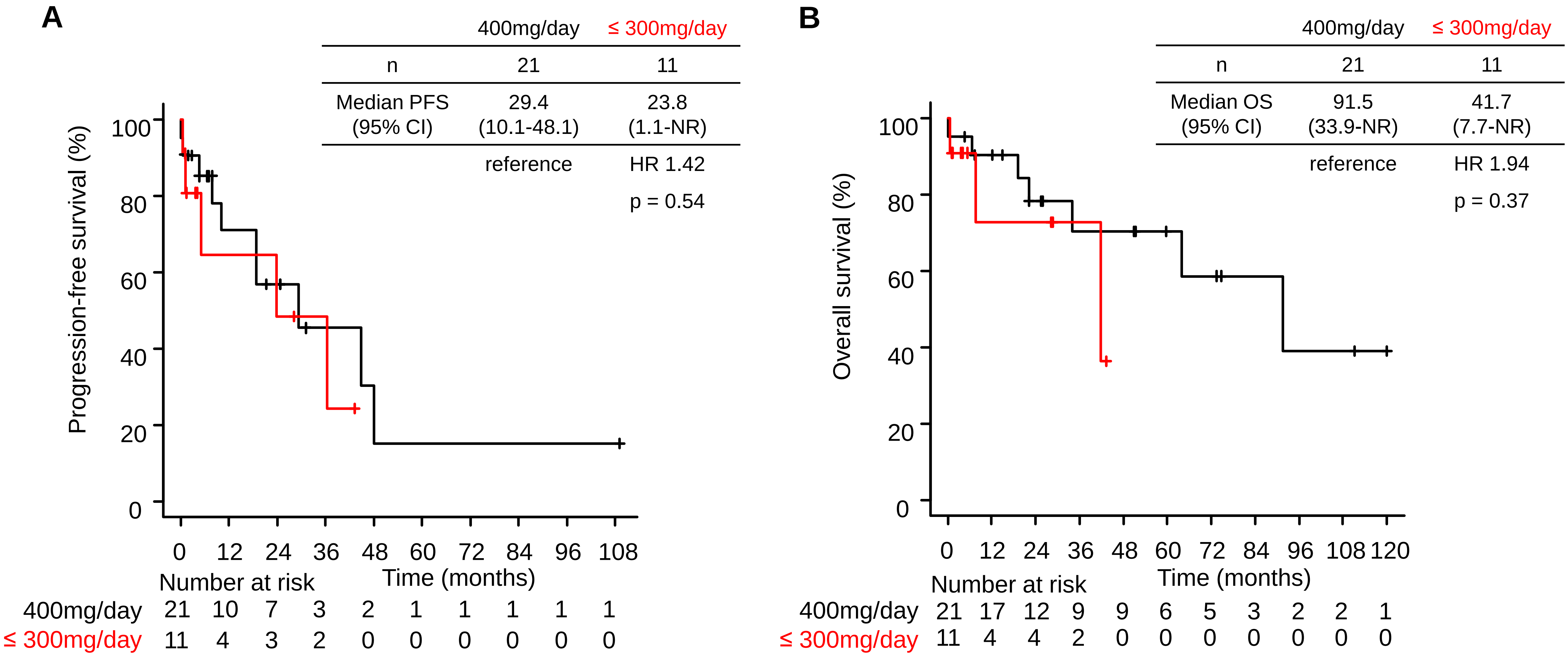

Supplement: Supplementary file 2 — Figure S1. Kaplan–Meier survival curves for initial dose of imatinib in elderly population. (A) Progression‐free survival. (B) Overall survival. [file CAM4-14-e71338-s002.pdf]
