# Supplementary material for: A Retrospective Analysis of the Efficacy and Safety of Imatinib for Advanced Gastrointestinal Stromal Tumor in Elderly Patients
Source: Cancer Med. 2025 Oct 31;14(21):e71338. doi: 10.1002/cam4.71338 (PMC12576807; doi:10.1002/cam4.71338)

Supplementary Figure 2. Comparison of the treatment duration before and after imatinib dose reduction

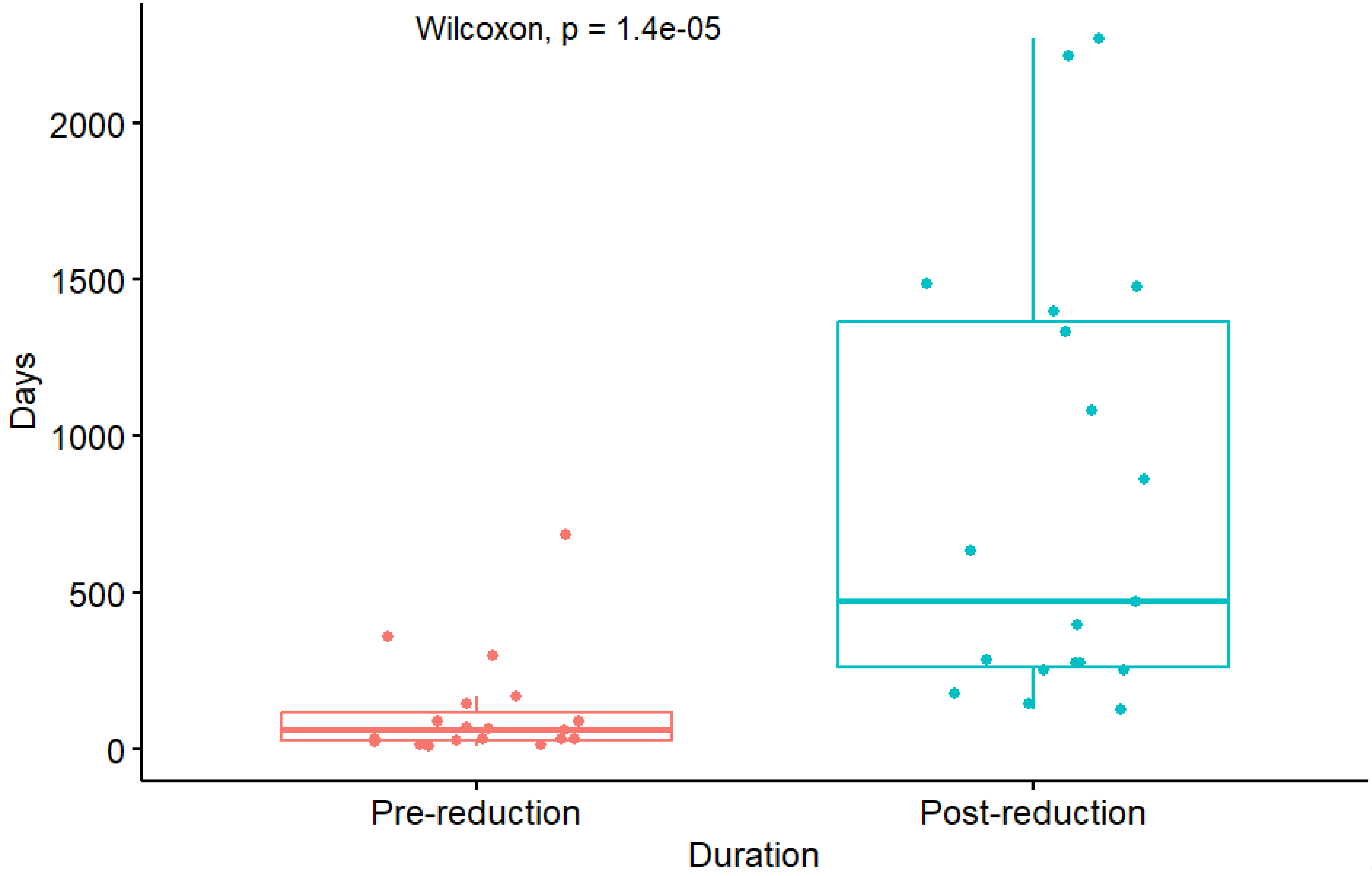

Supplement: Supplementary file 3 — Figure S2. Comparison of the treatment duration before and after imatinib dose reduction. [file CAM4-14-e71338-s003.pdf]
